# Molecular Mechanisms of Cigarette Smoke-Induced Proliferation of Lung Cells and Prevention by Vitamin C

**DOI:** 10.1155/2011/561862

**Published:** 2011-05-31

**Authors:** Neekkan Dey, Dhruba J. Chattopadhyay, Indu B. Chatterjee

**Affiliations:** Department of Biotechnology and Dr. B. C. Guha Centre for Genetic Engineering and Biotechnology, Calcutta University College of Science, 35 Ballygunge Circular Road, Kolkata 700019, India

## Abstract

Lung cancer is the leading cause of cancer dearth. Cigarette smoking is the strongest risk factor for developing lung cancer, which is conceivably initiated by proliferation. Here, we show that low concentration of aqueous extract of cigarette smoke (AECS) causes excessive proliferation of human lung epithelial cells (A549) without any apoptotic cell death. The causative factor responsible for AECS-induced proliferation has been identified as p-benzoquinone (p-BQ). Coimmunoprecipitation and immunoblot experiments indicate that p-BQ binds with epidermal growth factor receptor (EGFR). However, in contrast to EGF, it causes aberrant phosphorylation of EGFR that lacks c-Cbl-mediated ubiquitination and degradation resulting in persistent activation of EGFR. This is followed by activation of Hras + Kras and the downstream survival and proliferative signaling molecules Akt and ERK1/2, as well as the nuclear transcription factors c-Myc and c-Fos. Vitamin C and/or antibody to p-BQ prevents AECS/p-BQ-induced proliferation of lung cells apparently by inactivating p-BQ and thereby preventing activation of EGFR and the downstream signaling molecules. The results suggest that vitamin C and/or antibody to p-BQ may provide a novel intervention for preventing initiation of lung cancer in smokers.

## 1. Introduction

Lung cancer is the leading cause of cancer death in the United States and throughout the world [1]. Cigarette smoking is the strongest risk factor for developing lung cancer. Smoking and exposure to environmental tobacco smoke account for 90% of lung cancer cases, and smokers have a 20-fold increased risk of death from lung cancer compared to nonsmokers [2]. However, the carcinogenic mechanisms of tobacco smoking are not well understood [3]. The most significant property of cancer cells is that they undergo excessive proliferation. Lung cancer arises after a series of progressive pathologic changes (preneoplastic lesions) that are initiated by proliferation (hyperplasia) [4]. In almost all instances, unregulated cell proliferation together with suppressed apoptosis constitutes the minimal common platform upon which all neoplastic progression occurs [5]. It has been proposed that increased proliferative activity is causally linked to carcinogenesis and tumor progression [6]. Experimental and theoretical support for the hypothesis that increased proliferation itself is a contributory factor to carcinogenesis stems mainly from studies with chemical carcinogens in rodent tumor models and mathematical modeling of tumor progression [7]. Clinical observations also suggest a possible contributory role of increased cell proliferation to genesis and/or progression of human cancers [7]. Since cigarette smoke (CS) causes lung cancer, it is expected that CS should promote cell division. In fact, preliminary observations indicate that hyperproliferation of cells occurs in response to smoke exposure [8–10]. However, the molecular mechanisms of CS-induced cell proliferation are yet to be known. This is particularly because cigarette smoke (CS) is a highly complex mixture containing about 4000 compounds, including carcinogens, free radicals, and long-lived radicals such as semiquinones [11, 12]. It is a conjecture whether one particular compound or a number of compounds in CS are responsible for proliferation of cells. We have isolated a major semiquinone from CS and characterized it as p-benzosemiquinone (p-BSQ) [13, 14]. p-BSQ is present in substantial amounts (100–200 *μ*g/cigarette) in smoke from all commercial cigarettes examined as well as Kentucky research cigarettes [15]. p-BSQ causes cytotoxicity and tissue damage through conversion to p-benzoquinone (p-BQ), which occurs by disproportionation and oxidation by transition metal-containing proteins [14, 16]. Here, we show that CS-induced proliferation of lung cells is completely prevented by antibody to p-BQ and that p-BQ in amounts derived from CS mimics CS-induced lung cell proliferation. Overexpression and/or hyperactivity of the epidermal growth factor receptor (EGFR), accompanied by several downstream cytoplasmic signal transducers, including Ras-MAPK cascade as well as the cell survivor factor Akt (protein kinase B) has been shown to play a causal role in the proliferation and progression of lung tumors [4]. Lemjabbar et al. observed that CS-induced cell proliferation was accompanied by phosphorylation (activation) of the epidermal growth factor receptor (EGFR) [9]. Abdelmohsen et al. showed that p-BQ induced activation of extracellular signal-regulated kinase ERK1/2 via the activation of EGFR [17]. Here, we demonstrate that p-BQ, apparently derived from p-BSQ of aqueous extract of CS (AECS), is responsible for AECS-induced cell proliferation via activation of EGFR, Ras, ERK1/2, and Akt as well as the transcription factors c-Myc and c-Fos. p-BQ is strongly inactivated by vitamin C, and earlier we had reported that CS-induced cytotoxicity and lung damage is prevented by vitamin C [18–22]. Here, we show that both antibody to p-BQ and vitamin C prevent AECS/p-BQ-induced proliferation of human lung cells apparently by inactivating p-BQ. 

## 2. Materials and Methods

### 2.1. Chemicals and Reagents

p-Benzoquinone (p-BQ) was procured from Merck and freshly crystallized before use. Benzo[a]pyrene (BP) was obtained from Fluka. 4-(Methylnitrosamino)-1-(3-pyridyl)-1-butanone (NNK) and N-nitrosonornicotine (NNN) were obtained from Toronto Research Chemicals Inc. Tyrphostin AG1478 was purchased from Sigma-Aldrich, USA. The “In Situ Cell Proliferation Kit, FLUOS” was obtained from Roche Applied Science, Germany. The Annexin V-FITC kit was purchased from BD Biosciences. The kit for protein estimation was purchased from Bio-Rad, USA and the Protein A-Sepharose CL-42 beads from GE Healthcare, USA. The chemiluminescence kit for immunoblot analysis was procured from Cell Signaling Technology, USA. All other chemicals were of analytical grade. The antibody to p-BQ, raised in rabbit after immunization with p-BQ-bovine serum conjugate, was supplied by Abexome Biosciences, Bangalore, India.

### 2.2. Cigarettes

All the experiments were performed using cellulose-acetate filter-tipped Kentucky reference cigarettes (3R4F) obtained from the University of Kentucky, College of Agriculture Reference Cigarette Program, Lexington, Kentucky, USA.

### 2.3. Preparation of Aqueous Extract of Cigarette Smoke (AECS) Solution

The method of preparation of AECS was so devised as to simulate the manner in which the respiratory tract lining fluid is exposed to CS during the process of smoking by humans [18]. Smoke from one cigarette was extracted with 1 mL of 50 mM potassium phosphate buffer, pH 7.4, filtered through 0.22 *μ*m Millipore filter and the pH adjusted to 7.4. The aqueous extract of CS (AECS) solution thus obtained was used immediately.

### 2.4. Measurement of p-Benzoquinone (p-BQ)

p-BQ was measured by HPLC as described before [14]. The column used was a LichroCART 350-4, RP-18 (5 *μ*m) (Merck). p-BQ was detected at 245 nm at the retention time of 4.75 min using a mobile solvent of methanol: water (90 : 10 v/v) at a flow rate of 0.5 mL/min. The limit of detection was 500 pg. 

### 2.5. Cell Culture

A549 human lung carcinoma cells were maintained in Hams F12 medium (GIBCO-BRL, USA) containing 10% fetal calf serum (GIBCO-BRL, USA), 100 units/mL penicillin, 100 *μ*g/mL streptomycin, and 4 mM glutamine/mL. L132 (normal human lung epithelial cells), Vero (African green monkey kidney cell line), and HepG2 (human liver cell line) cells were maintained in Dulbecco's Modified Eagle Medium (GIBCO-BRL, USA), containing 10% fetal calf serum (GIBCO-BRL, USA), 100 units/mL penicillin, 100 *μ*g/mL streptomycin, and 4 mM glutamine/mL. All the cells were obtained from National Centre for Cell Sciences (NCCS Pune, India). The cells were grown at 37°C in a humified incubator maintained in an atmosphere of 95% air and 5% CO_2_.

### 2.6. Cytotoxicity Assay

The cytotoxicity of AECS, p-BQ, NNN, NNK, and BP was evaluated by the 3-(4,5-dimethylthiazol-2-yl)-2,5-diphenyl tetrazolium bromide (MTT) assay, as described earlier [15]. Briefly, after treatment with different compounds, the culture medium was replaced with serum-free medium containing 0.5 mg/mL MTT, and cultures were incubated for an additional 3 hr. The blue MTT formazan thus formed was dissolved in DMSO, and the absorbance values were measured at 560 nm in a UV-VIS spectrophotometer (Shimadzu UV-2540).

### 2.7. Cell Cycle Analysis by Flow Cytometry

To estimate the percentage of cells at different stages of cell cycle, cultured cells (3 × 10^6^), after specified treatment with AECS/p-BQ for 1 hr, were washed with PBS solution and incubated in fresh culture medium for 12 hr. Cell cycle analysis was performed by propidium iodide (PI) according to manufacturer's protocol and analyzed using the FACS Calibur-Cell Quest software. A total of 10,000 events were acquired, and a histogram plot of FL2-H was recorded.

### 2.8. Assessment of Apoptosis by Flow Cytometry

To estimate the percentage of cells undergoing apoptosis, cultured cells (3 × 10^6^), after specified treatment with AECS for 1 hr, were washed with PBS solution (Hyclone, Thermo Scientific) and incubated in fresh culture medium for 12 hr. Apoptosis was assessed by Annexin V and propidium iodide (PI) (Becton Dickinson) according to manufacturer's protocol and analyzed using the FACS Calibur-Cell Quest software (Becton Dickinson) as described earlier [15]. A total of 10,000 events were acquired, and dual parameter dot plot of FL2-H (*x*-axis; PI-fluorescence, linear scale) versus FL1-H (*y*-axis; Annexin V-FITC-fluorescence, linear scale) was recorded. 

### 2.9. Lysate Preparation, Immunoprecipitation, and Immunoblotting

Lysate preparation and protein immunoprecipitation were performed as described by Bao et al. [23]. After treatment, cells were extracted in solubilization buffer containing 50 mM Tris, pH 7.5; 150 mM NaCl; 10% glycerol; 1% Nonidet P-40; 1 mM EDTA; Protease Inhibitor Cocktail (Sigma); Phosphatase Inhibitor Cocktail (Sigma). Lysates were cleared by centrifugation at 20,000 g for 10 min at 4°C, and the total protein concentration was estimated. Protein (400 *μ*g) in the supernatant was immunoprecipitated by overnight incubation with 4 *μ*g anti-EGFR antibody (Cell Signaling Technology, USA) at 4°C, followed by Protein A-Sepharose CL-42 (GE Healthcare) precipitation for 3 hr at 4°C. Immunoprecipitates were washed 3 times with HNTG buffer containing 20 mM HEPES, pH 7.5; 150 mM NaCl; 0.1% Triton X-100; 10% glycerol, resolved by SDS-PAGE, and transferred to PVDF membrane. Membranes were blocked for 1 hr in Tris-buffered saline, pH 7.5; containing 0.5% Tween 20 and 5% nonfat milk (Bio-Rad), incubated overnight at 4°C with primary antibody (1 : 1000), followed by 1 hr incubation at room temperature with 1 : 3000 dilution of HRP-conjugated secondary antibody (Cell Signaling Technology, USA). Immunoreactive protein bands were detected by chemiluminescence. Blotting antibodies used were anti-EGFR, anti-p-BQ, anti-p53, anti-phospho-p53, anti-Caspase 3, anticleaved Caspase 3, anti-Akt, anti-phospho-Akt, anti-phosphotyrosine-845, anti-phosphotyrosine-1045, anti-phosphotyrosine-1068, anti-phosphotyrosine-1086, anti-ERK1/2, anti-phospho-ERK1/2, anti-c-Fos, anti-c-Cbl, antiubiquitin(UbC3) (Cell Signaling Technology, USA), antiphosphotyrosine PY20, anti-phosphotyrosine-1173, anti-*β* actin (Santa Cruz Biotechnology, USA), anti-HRAS + KRAS, anti-c-Myc, and anti-phospho-c-Myc (phospho T58+S62) (abcam, UK).

### 2.10. Detection of Reactive Oxygen Species (ROS) Production

Prior to treatment, cells were incubated for 30 min with 10 *μ*M 2′,7′-dichlorodihydrofluorescein diacetate (H_2_DCFDA) (Sigma-Aldrich). Fluorescent images were captured using confocal laser scanning microscope (LSM 510 META, Carl Zeiss).

### 2.11. Cell Proliferation Assay

Cell proliferation was assessed using the “In Situ Cell Proliferation Kit, FLUOS” (Roche Applied Science, Germany), according to the manufacturer's protocol. Briefly, after treatment the cells were incubated in serum-free medium containing 10 *μ*M BrdU Labeling Solution for 5 hr at 37°C. Cells were washed with PBS and fixed for 30 min at 4°C in fixative solution, containing 50 mM glycine (pH 2.0) in 70% (V/V) ethanol. Cells were then permeabilized for 20 min at room temperature with denaturation solution, containing 4 M HCl, followed by subsequent PBS washes until the pH reached above 6.5. To block nonspecific binding, cells were then incubated for 10 min at room temperature in incubation buffer, containing PBS, 0.5% BSA, and 0.1% Tween 20. Cells were then incubated with anti-BrdU-FLUOS antibody for 45 min at 37°C in a humid chamber. After incubation, cells were washed with PBS and analyzed by flow cytometry using the FACS Calibur-Cell Quest software (Becton Dickinson). A total of 10,000 events were acquired, and a histogram plot of FL1-H was recorded.

### 2.12. Statistical Analysis

All values are expressed as mean ± SD. Statistical significance was carried out using one-way ANOVA. The *P* values were calculated using appropriate *F*-tests. Difference with *P* values <.05 was considered significant.

## 3. Results and Discussion

### 3.1. Proliferation of Human Lung Epithelial Cells (A549) by AECS/p-BQ

**Figure 1:**
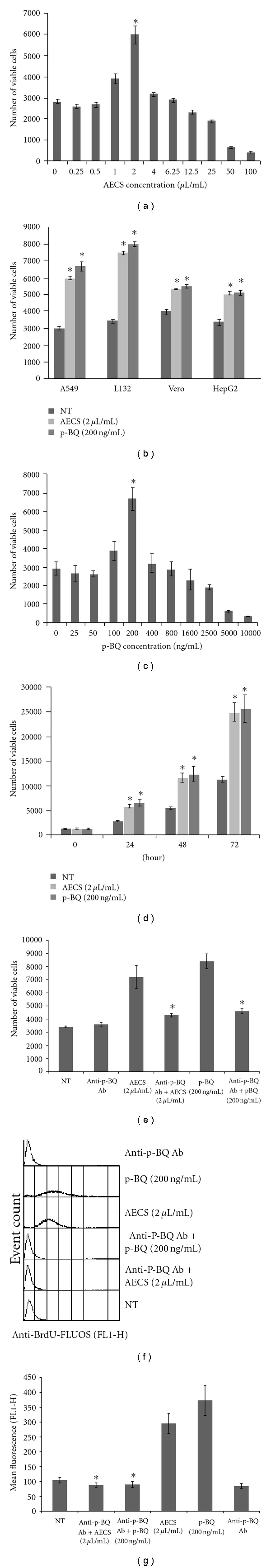
AECS/p-BQ-induced proliferation of human lung epithelial cells (A549) and its prevention by anti-p-BQ antibody. Except in (d), all the treatments were made in 24 hr. Effect of concentration gradient of AECS on proliferation and death of A549 cells in culture, as determined by MTT cytotoxicity assay (a). AECS (2 *μ*L/mL) or p-BQ (200 ng/mL)-induced proliferation in other cell lines, namely. L132, Vero, and HepG2 (b). Effect of concentration gradient of p-BQ on proliferation and death of A549 cells in culture, as determined by MTT cytotoxicity assay (c). Treatment with AECS (2 *μ*L/mL) or p-BQ (200 ng/mL) results in continued proliferation for 24–72 hr (d). AECS/p-BQ-induced proliferation is prevented by anti-p-BQ antibody, as evidenced by MTT assay (e). The inhibitory role of anti-p-BQ antibody on the proliferation of A549 cells as determined by the incorporation of BrdU using flow cytometry assay (f, g). All data are depicted as the mean ± SD for three independent experiments (∗ indicates significant difference, *P* < .05 in comparison to nontreated control (a, b, c, d); and in comparison to AECS and p-BQ, resp. (e, g)).

Using MTT assay here we show that whereas low concentration of aqueous extract of cigarette smoke (AECS) induces proliferation of human lung epithelial cells in culture (A549), high concentrations lead to cell death (Figure 1(a)). The optimum AECS concentration that causes maximum cell proliferation is about 2 *μ*L/mL. The proliferation is not restricted to A549 cells; it also occurs in other cell lines, such as L132 (normal human lung epithelial cells), Vero (African green monkey kidney cell line), and HepG2 (human liver cell line) (Figure 1(b)). The AECS-induced proliferation is mimicked by the amount of p-BQ (200 ng/mL) produced in the culture medium from 2 *μ*L/mL of AECS (Figure 1(c)). The amount of p-BQ formed from AECS in the incubation mixture was determined by HPLC. Like that observed with AECS, high concentrations of p-BQ cause cell death (Figure 1(c)). A single treatment with AECS (2 *μ*L/mL) or p-BQ (200 ng/mL) results in continued proliferation for 24–72 hr (Figure 1(d)).The proliferation by either AECS or p-BQ is completely prevented by antibody to p-BQ (Figure 1(e)). The inhibitory role of anti-p-BQ antibody on the proliferation of A549 cells has also been confirmed by the incorporation of BrdU using flow cytometry analysis (Figures 1(f) and 1(g)). The results indicate that p-BQ derived from AECS is responsible for AECS-induced proliferation of the lung cells. The AECS used was prepared from Kentucky research cigarettes (3R4F). Similar results were obtained by AECS prepared from a commercial cigarette (Wills Navy Cut, India; results not shown), indicating that the observations were not specific to Kentucky research cigarettes.

p-BQ is not present in CS, but is formed from p-benzosemiquinone (p-BSQ), a long-lived semiquinone present in substantial amounts (100–200 *μ*g/cigarette) in smoke from Kentucky research cigarettes as well as a number of commercial cigarettes examined [15]. p-BSQ is present exclusively in the tar phase of CS and is extracted in the AECS [12, 14]. p-BSQ is converted to p-BQ by disproportionation (2 p-BSQ→p-BQ + HQ) [16], as well as oxidation by transition metal (Fe^3+^, Cu^2+^) containing proteins (p-BSQ→p-BQ) [14]. The method of preparation of AECS was so devised as to simulate the manner in which the respiratory tract lining fluid is exposed to CS during the process of smoking by humans [18]. Cigarette tar is continually being deposited in the lungs of smokers, and these lungs are continually bathed in an aqueous solution that can solubilize and transport the water soluble components of the tar [12]. p-BSQ present in cigarette tar can be extracted into aqueous solutions and thus would be in solutions bathing a smoker's lung [12]. In CS solution produced in the lungs, p-BSQ would be converted to p-BQ and induce proliferation of cells. This is supported by the observation that CS causes cell proliferation in the lungs of rat in vivo [8]. 

### 3.2. Cell Cycle Analysis of the Proliferating Cells

**Figure 2:**
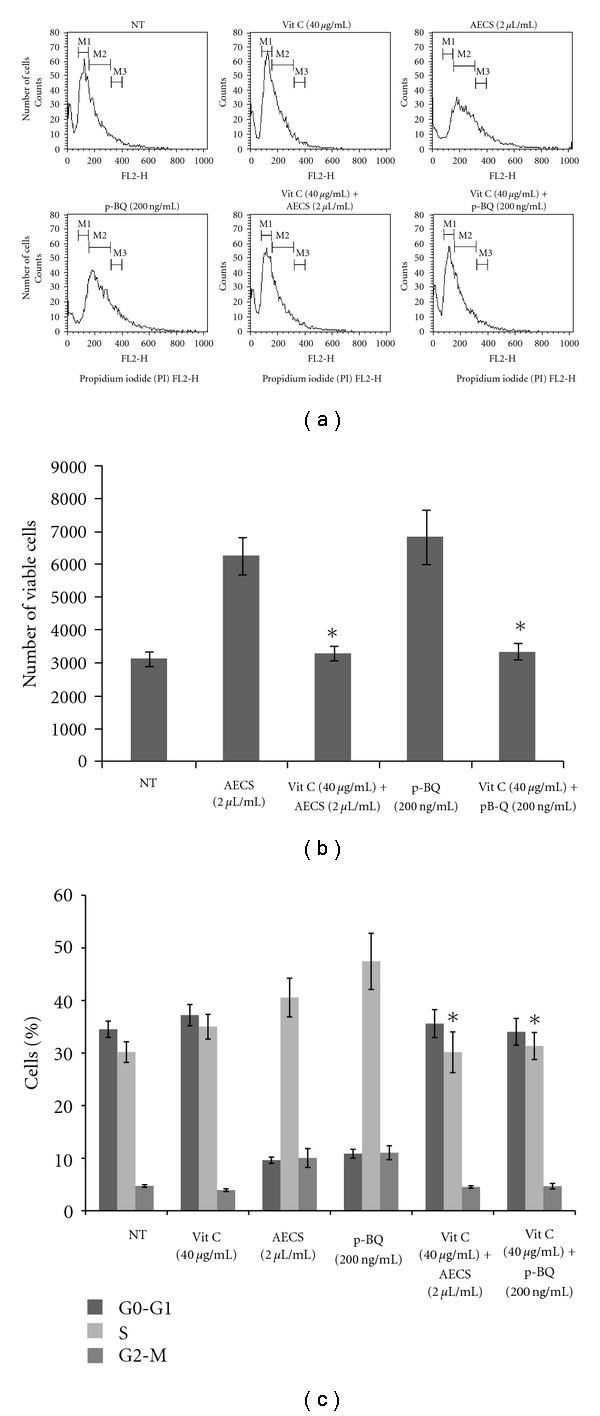
Cell cycle analysis of proliferating A549 cells. Histogram plot (Event count versus FL2-H) of AECS/p-BQ-induced proliferation of A549 cells and its prevention by vitamin C as determined by propidium iodide (PI) staining followed by flow cytometry (a). Bar diagram showing mean fluorescence of PI (FL2-H) in cells either nontreated (NT) or exposed to 2 *μ*L/mL AECS or 200 ng/mL p-BQ for 24 hr and its prevention by vitamin C (40 *μ*g/mL) (b). Bar diagram showing the relative cell distribution during various phases of the cell cycle after exposure to 2 *μ*L/mL AECS or 200 ng/mL p-BQ (c). Data are means ± SD for three independent experiments (∗ in (b) and (c) indicates significant difference, *P* < .05 in comparison to AECS and p-BQ treatment).

The rate of cell proliferation within any population of cells depends on three parameters: (a) the rate of cell division, (b) the fraction of cells within the population undergoing cell division, and (c) the rate of cell loss from the population due to terminal differentiation or cell death. Failure to regulate these functions properly results in an altered phenotype and cancer [19]. Here, we have performed the cell cycle analyses using propidium-iodide (PI) staining followed by flow cytometry.  Figure 2(a) shows the histogram plot of A549 cells either nontreated (NT) or exposed to 2 *μ*L/mL AECS or 200 ng/mL p-BQ for 24 hr. The mean fluorescence of 2 *μ*L/mL AECS or 200 ng/mL p-BQ-treated cells was significantly higher (*P* < .05) than that of the nontreated cells. However, pretreatment with 40 *μ*g/mL vitamin C for 15 min completely reduces the mean fluorescence (Figure 2(b)). In addition to the relative cellular DNA content, the cell distribution during the various phases of the cell cycle was also determined (Figure 2(c)). Three distinct phases were recognized in the AECS/p-BQ-induced proliferating cell population: the G0-G1 (Region “M1” in Figure 2(a)), S or the DNA synthesis phase (Region “M2” in Figure 2(a)), and the G2-M phase (Region “M3” in Figure 2(a)). Also, the percentages of cells occupying the different phases of the cell cycle were calculated (Figure 2(c)). Figure 2(c) shows that compared to the nontreated cells (30.2%), cells treated with 2 *μ*L/mL AECS or 200 ng/mL p-BQ have higher number of cells (40.55% or 47.45% of the total cell population, resp.) in the S-phase, indicating markedly higher rate of DNA synthesis. However, pretreatment with 40 *μ*g/mL vitamin C for 15 min prior to treatment with 2 *μ*L/mL AECS or 200 ng/mL p-BQ reduces the percentage of cells in S-phase to 30.15% and 31.35%, respectively (Figures 2(a), 2(b), and 2(c)), indicating prevention of higher rate of DNA synthesis.

### 3.3. Vitamin C Prevents AECS/p-BQ-Induced Proliferation of Cells

**Figure 3:**
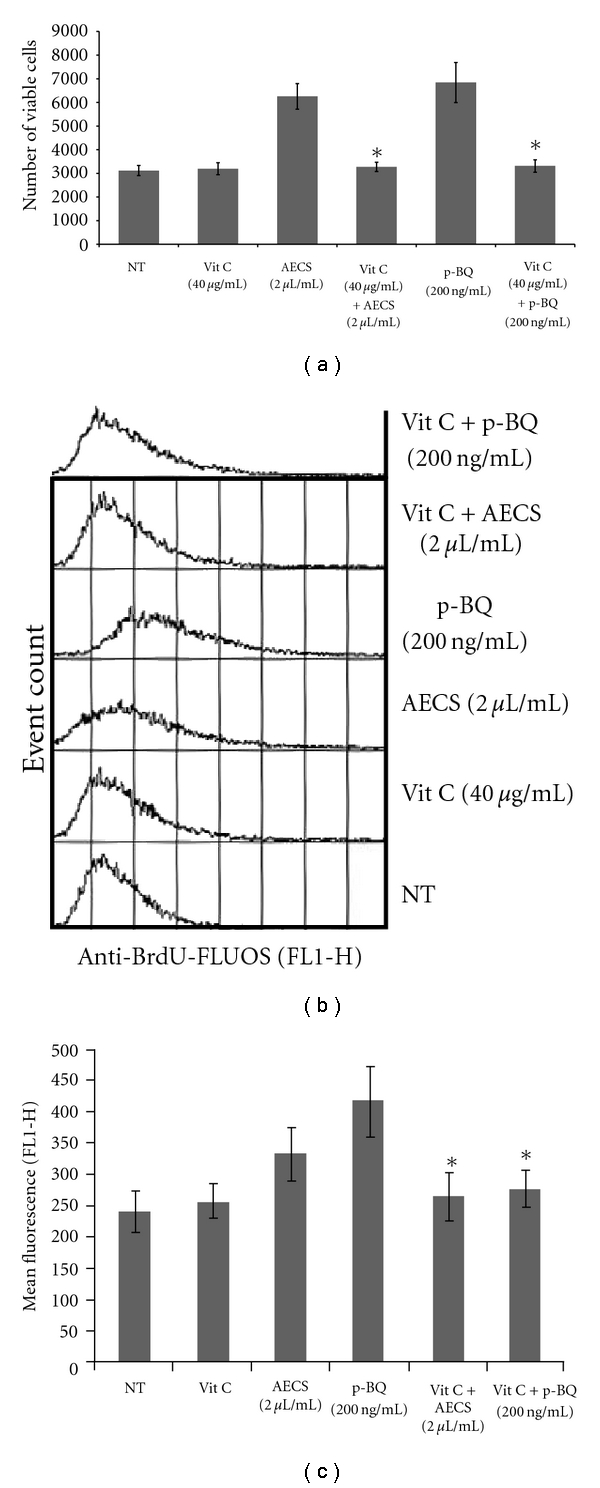
Vitamin C prevents AECS/p-BQ-induced proliferation of cells. AECS/p-BQ-induced proliferation is prevented by vitamin C (40 *μ*g/mL), as evidenced by MTT assay (a), as well as BrdU-incorporation assay (b and c). Serum-starved A549 cells (2000 cells/well) grown on 96-well tissue culture plates were either nontreated (NT) or exposed to 2 *μ*L/mL AECS or 200 ng/mL p-BQ for 24 hr with or without vitamin C (40 *μ*g/mL) pretreatment in serum-free medium for 15 min (a, b, and c). Data represent means ± SD for three independent experiments (∗ indicates significant difference, *P* < .05 in comparison to AECS (2 *μ*L/mL) and p-BQ (200 ng/mL), resp.).

We have previously shown that CS produces toxicity and tissue damage only in marginal vitamin C-deficient guinea pigs, but not in vitamin C-sufficient ones [14, 18, 20]. We had also shown that a moderately large dose of vitamin C prevents CS-induced toxicity, apparently by reducing and inactivating p-BQ (14). This is because vitamin C (*E*° = +0.08 V) strongly reduces p-BQ (*E*° = +0.71 V) to less toxic hydroquinone and thereby inactivates p-BQ. Here, we show that p-BQ derived from AECS causes proliferation of lung cells in serum-free medium (Figure 1), which is essentially free of vitamin C. MTT assay also indicates that 40 *μ*g/mL vitamin C prevents AECS/p-BQ-induced proliferation of A549 cells (Figure 3(a)). At this concentration, vitamin C does not have any effect on the growth of nontreated (NT) cells in the absence of AECS/p-BQ (data not shown). The inhibitory role of vitamin C has been confirmed by BrdU incorporation, as evidenced by flow cytometry assay (Figures 3(b) and 3(c)).

The role of vitamin C in the prevention and treatment of cancer has a long and controversial history. Although there has been a paucity of human studies using vitamin C to treat already existing cancer, there is considerable epidemiological evidence pointing to the benefits of vitamin C in the prevention of a number of types of cancer, including lung cancer [24, 25]. Almost 90% of lung cancer is due to cigarette smoking [2]. We had shown that CS consumes vitamin C [18]. This would corroborate the observations by other researchers that lung cancer patients usually suffer from hypovitaminosis C [25]. Several clinical trials of cancer and vitamin C demonstrated remarkable tolerance and safety for high dose of vitamin C in patients [26].

**Figure 4:**
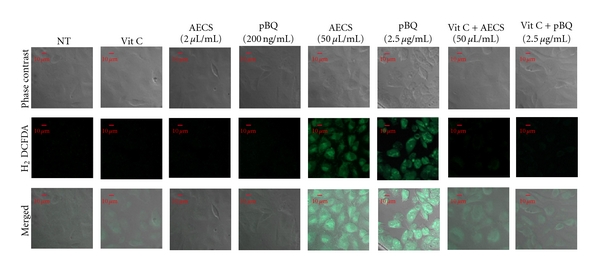
Effect of low or high concentration of AECS/p-BQ on reactive oxygen species (ROS) production in cultured A549 cells and its prevention by vit C. Serum-starved A549 cells grown on coverslips were either nontreated (NT) or exposed to 2 *μ*L/mL AECS, 50 *μ*L/mL AECS, 200 ng/mL p-BQ or 2.5 *μ*g/mL p-BQ for 1 hr with or without vitamin C (40 *μ*g/mL) pretreatment in serum-free medium for 15 min. After treatment, cells were incubated in fresh media containing H_2_DCFDA for 30 min and PBS washed twice, and fluorescent images were captured.

**Figure 5:**
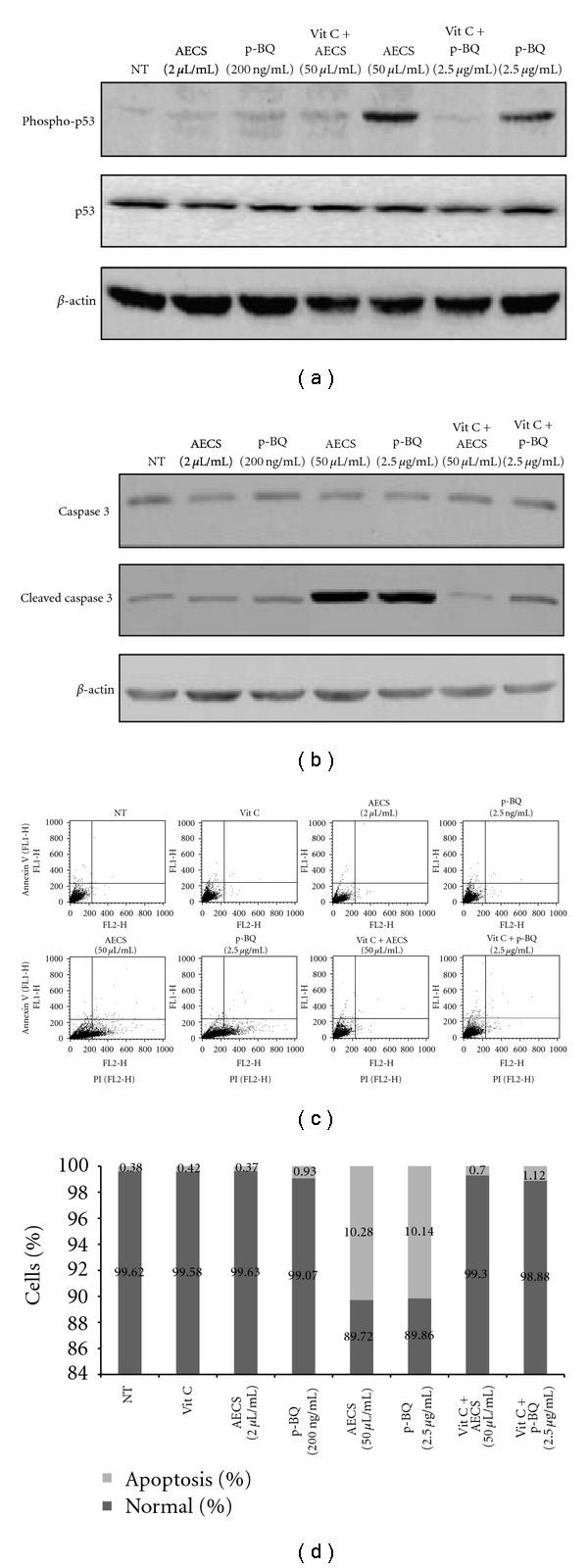
Status of p53, phospho-p53, caspase 3, cleaved-caspase 3 and apoptosis in cultured A549 cells exposed to low or high concentration of AECS/p-BQ. The figure represents immunoblots of phosphorylated p53 and p53 (a) and caspase 3 and cleaved caspase 3 (b). Cell lysate of A549 cells were either nontreated (NT) or exposed to AECS (2 *μ*L or 50 *μ*L/mL) or p-BQ (200 ng or 2.5 *μ*g/mL) for 1 hr followed by incubation in serum containing media for 12 hr. Vitamin C (40 *μ*g/mL) pretreatment of cells in serum-free media for 15 min prevented AECS/p-BQ-induced activation of p53 or cleavage of caspase 3 (a, b). *β*-actin was used as the loading control. Effect of low or high concentrations of AECS/p-BQ on apoptosis in cultured A549 cells and its prevention by vitamin C as evidenced by flow cytometry (c). A549 cells were grown on 60 mm culture plates and were gradually serum starved for 3 days to synchronize the cells. Then the cells were either nontreated (NT) or exposed to 2 *μ*L/mL or 50 *μ*L/mL AECS for 1 hr, 200 ng/mL or 2.5 *μ*g/mL p-BQ for 1 hr, with or without 40 *μ*g/mL vit C pretreatment in serum-free medium for 15 min. After treatment, cells were incubated in fresh media containing serum for 12 hr, followed by Annexin V-PI assay. Bar graphs show the percentage of normal and apoptotic cells after respective treatments, as evidenced by flow cytometry (d). The numbers within the bars represent percentage of normal and apoptotic cells.

It has been shown above that in contrast to low concentration, high concentration of AECS/p-BQ (50 *μ*L/mL AECS or 2.5 *μ*g/mL p-BQ) results in cell death (Figure 1(a)). The death is apparently caused by oxidative stress and apoptosis. The oxidative stress has been evidenced by the formation of ROS (Figure 4) and apoptosis by the phosphorylation of p53 (Figure 5(a)) and activation (cleavage) of caspase 3 (Figure 5(b)). Apoptosis was supported by Annexin V/PI assay using flow cytometry (Figures 5(c) and 5(d)). Vitamin C prevents cell death apparently by preventing oxidative stress (Figure 4) and apoptosis (Figure 5). No such oxidative stress or apoptosis was observed with low concentration of AECS (2 *μ*L) or p-BQ (200 ng) that induced proliferation of cells (Figures 4 and 5).

### 3.4. Effects of NNK, NNN, and BP on Cell Proliferation

**Figure 6:**
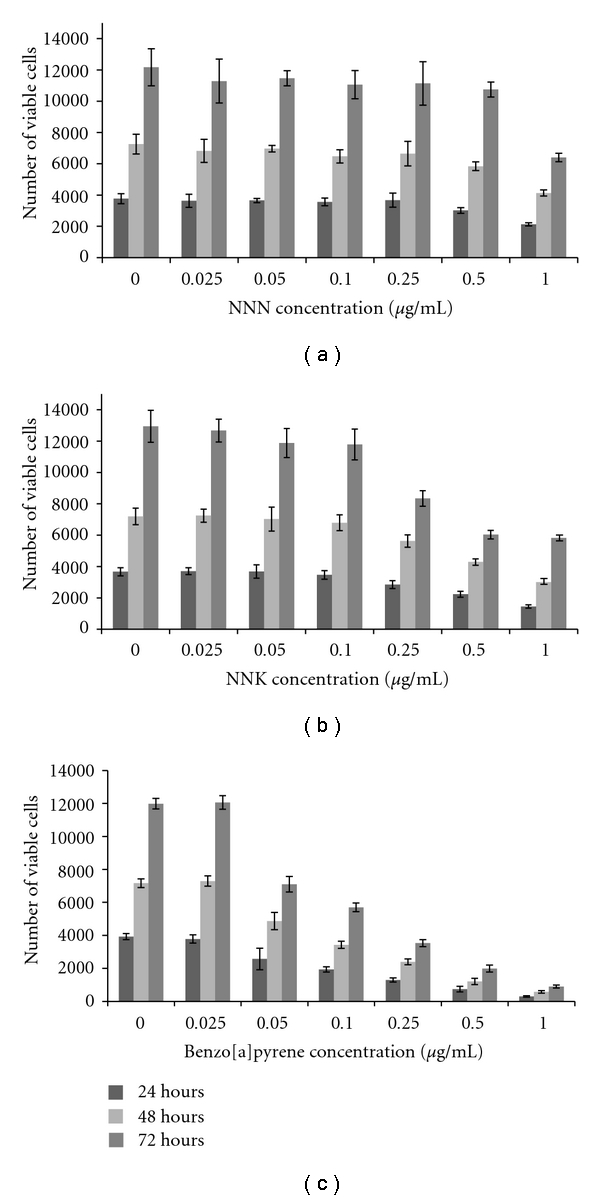
Effects of concentration gradient of NNN (a), NNK (b), and BP (c) on the growth of cultured A549 cells after 24, 48, and 72 hr as evidenced by MTT assay. Serum-starved A549 cells (2000 cells/well) grown on 96-well tissue culture plates were either nontreated (0) or exposed to 0.025, 0.05, 0.1, 0.5, or 1 *μ*g/mL NNN (a), NNK (b), and BP (c) for 24, 48, and 72 hr. After treatment, MTT cytotoxicity assay was performed. All data are depicted as the means ± SD for four independent experiments.

Cigarette smoke is a complex mixture of 4000 compounds containing carcinogens, including polycyclic aromatic hydrocarbons (PAHs) and nitrosamines. Among PAH, the most extensively studied is benzo[a]pyrene (BP) and among nitrosamines, 4-(Methylnitrosamino)-1-(3-pyridyl)-1-butanone (NNK) and N-nitrosonornicotine (NNN) [11]. Compared to p-BSQ (100–200 *μ*g/cigarette) [15], the concentrations of these carcinogens in smoke from one cigarette are very low: BP, 20–40 ng; NNK, 80–770 ng; NNN, 1.1–2.9 *μ*g [11, 27]. These carcinogens produce tumor in rodents only at very high doses [11]. Here, we show that under the experimental conditions, NNN, NNK, and BP do not cause any proliferation of A549 cells (Figures 6(a), 6(b), and 6(c)). At high concentrations, the carcinogens are rather inhibitory to the growth of the cells. Moreover, none of them has any synergistic effect on the proliferation of A549 cells by AECS or p-BQ (data not shown). Although BP, NNK, and NNN do not induce proliferation of cells, but they may exert their carcinogenic effects on p-BQ-induced cell proliferation. Proliferation (cell division) triggers mitotic recombination, gene conversion, and nondisjunction. The time interval for DNA repair during mitosis is short. The DNA is also transiently not base paired or bound to histones, therefore making it more sensitive to chance of adduct formation with DNA and mutation by BP, NNK, and NNN, ultimately leading to carcinogenesis. 

### 3.5. AECS/p-BQ-Induced Cell Proliferation Occurs via the Activation of EGFR That Is Prevented by Vitamin C

**Figure 7:**
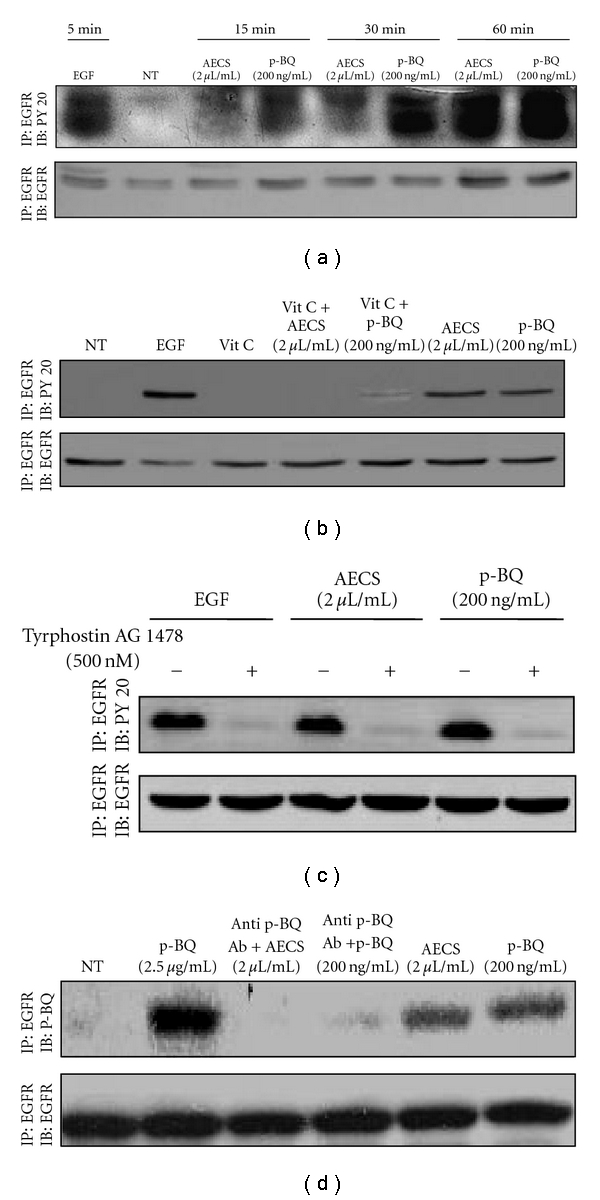
p-BQ mimics AECS in activating EGFR in a time-dependent manner. Serum-starved A549 cells were nontreated (NT) or exposed to 100 ng/mL EGF for 5 min, 2 *μ*L/mL AECS or 200 ng/mL p-BQ for 15 min, 30 min, or 1 hr, respectively (a). Vitamin C (40 *μ*g/mL) pretreatment of A549 cells for 15 min in serum-free media completely prevented AECS/p-BQ-induced EGFR activation (b). 500 nM Tyrphostin AG1478 (EGFR inhibitor) pretreatment of A549 cells for 15 min in serum-free media completely prevented activation of EGFR by 100 ng/mL EGF, 2 *μ*L/mL AECS, or 200 ng/mL p-BQ (c). Anti-p-BQ antibody prevented AECS/p-BQ-induced EGFR activation (d). Cells were lysed, and the EGFR was immunoprecipitated (IP) from the cell lysates using anti-EGFR antibody. Immunoprecipitated proteins were separated by SDS-PAGE, transferred to PVDF membrane, and immunoblotted (IB) with either antiphosphotyrosine (PY20) antibody (a, b, c) or with anti-p-BQ antibody (d).

Epidermal growth factor receptor (EGFR) is composed of an extracellular ligand binding domain, a transmembrane domain, and an intracellular tyrosine kinase (receptor tyrosine kinase, RTK)) domain. Activation of the receptor leads to an intracellular signaling cascade that controls cellular proliferation and differentiation. Ligands for these receptors, most importantly epidermal growth factor (EGF) and transforming growth factor-*α* (TGF-*α*), bind to the extracellular domain resulting in receptor dimerization and autophosphorylation of the intracellular receptor tyrosine kinase (RTK) domain, leading to downstream signaling, including the activation of *ras*, *raf*, mitogen-activated protein kinase (MAPK), phosphatidyl-3 kinase (PI3K/Akt), and ERK1/2. These molecules are linked to cell growth, proliferation, motility, and survival [28]. Previous studies revealed that EGFR is activated (phosphorylated) in a dose- and time-dependent manner when exposed to CS solution [29]. However, CS is a highly complex mixture, and the component(s) of cigarette smoke solution responsible for cell proliferation has not been known. Using human lung epithelial cells (A549), here we show that p-BQ (200 ng/mL) derived from AECS (2 *μ*L/mL) is responsible for AECS-induced phosphorylation of EGFR. (Figure 7(a)). The activation (phosphorylation) of EGFR by EGF is mediated by noncovalent interactions, and the phosphorylation appears to be high after 5 min (Figure 7(a)), which decays after 15 min as shown by others [29]. On the other hand, p-BQ derived from AECS covalently binds with the extracellular domain of EGFR tentatively by Michael addition with Lys residue [30] and activates the EGFR constitutively. In this case, the phosphorylation by p-BQ starts at about 15 min after treatment and persists for 1 hr, indicating prolonged activation of EGFR. It is reported that constant activation of EGFR results in uncontrolled cell division—a predisposition for cancer, including lung cancer [31, 32]. This would indicate that p-BQ may be a risk factor for the initiation of CS-induced lung cancer. Given that more than 80% of nonsmall cell lung carcinomas (NSCLCs) express EGFR [2], EGFR has become an important therapeutic target for the treatment of these tumors. This led to the development of EGFR inhibitors for anticancer treatment [33, 34]. The most common approaches use either monoclonal antibodies that competitively bind to the extracellular domain or small molecules targeting the intracellular RTK domain. Vitamin C inactivates p-BQ, and we have shown that vitamin C pretreatment of cells prior to AECS/p-BQ exposure completely prevents EGFR activation (Figure 7(b)). This would suggest that intake of vitamin C would prevent lung cell proliferation and initiation of carcinogenesis in smokers. Vitamin C does not prevent EGF-induced phosphorylation of EGFR (data not shown). In order to confirm that AECS/p-BQ induced activation of EGFR, we performed the aforesaid experiment in the presence of 500 nM AG1478 (Tyrphostin, EGFR kinase inhibitor). The results (Figure 7(c)) indicate that 500nM AG1478 completely prevents activation of EGFR by 100 ng/mL EGF, 2 *μ*L/mL AECS, or 200 ng/mL p-BQ. In a separate coimmunoprecipitation experiment, we show that anti-p-BQ antibody prevents AECS-induced EGFR activation (Figure 7(d)). This would indicate that antibody to p-BQ might also be a candidate for the prevention of the initiation of cell proliferation and lung cancer in smokers.

### 3.6. AECS/p-BQ Exposure Results in Aberrant Phosphorylation of the EGFR

**Figure 8:**
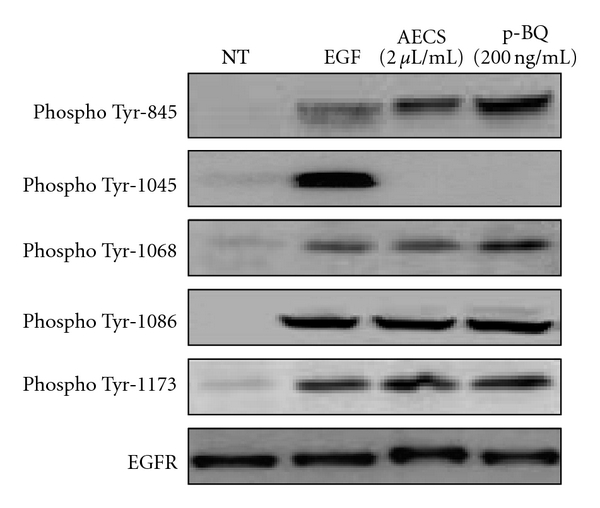
AECS/p-BQ exposure results in aberrant phosphorylation of the EGFR. Serum-starved A549 cells were either nontreated (NT) or exposed to 100 ng/mL EGF for 5 min, 2 *μ*L/mL AECS or 200 ng/mL p-BQ for 1 hr. Cells were lysed, and the EGFR was immunoprecipitated (IP) from the cell lysates using anti-EGFR antibody. Immunoprecipitated proteins were separated by SDS-PAGE, transferred to PVDF membrane, and immunoblotted (IB) with indicated EGFR phosphotyrosine-specific antibodies or anti-EGFR antibodies.

EGFR dimerization stimulates its intrinsic intracellular protein-tyrosine kinase activity. As a result, autophosphorylation of several tyrosine (Y) residues in the C-terminal domain of EGFR occurs. These include Y845, Y1045, Y1068, Y1148, and Y1173 [35]. This autophosphorylation elicits downstream activation and signaling by several other downstream signaling proteins that initiate several signal transduction cascades, principally the MAPK, Akt, and JNK pathways, leading to DNA synthesis and cell proliferation. Such proteins modulate phenotypes including cell proliferation [36]. Earlier studies indicate that CS exposure resulted in the aberrant phosphorylation of the EGFR [29]. Because p-BQ mimics AECS in activating the EGFR, we wanted to see whether p-BQ also causes aberrant phosphorylation of EGFR. Immunoblot analyses of EGFR from A549 cells exposed to 100 ng/mL EGF for 5 min, 2 *μ*L/mL AECS for 1 hr, or 200 ng/mL p-BQ for 1 hr show that AECS/p-BQ exposure results in similar aberrant phosphorylation pattern that is distinctly different from EGF exposure (Figure 8). After incubation with AECS/p-BQ, Tyr-845 is hyperphosphorylated, but Tyr-1045 is not phosphorylated. With EGF exposure, Tyr-1045 is strongly phosphorylated, whereas Tyr-845 is phosphorylated to a much lesser extent. The pattern of phosphorylation of Tyr-1068, 1086, and 1173 appears to be almost similar irrespective of treatment with EGF, AECS, and p-BQ.

### 3.7. EGFR Exposed to AECS/p-BQ Cannot Bind c-Cbl and Is Not Ubiquitinated

**Figure 9:**
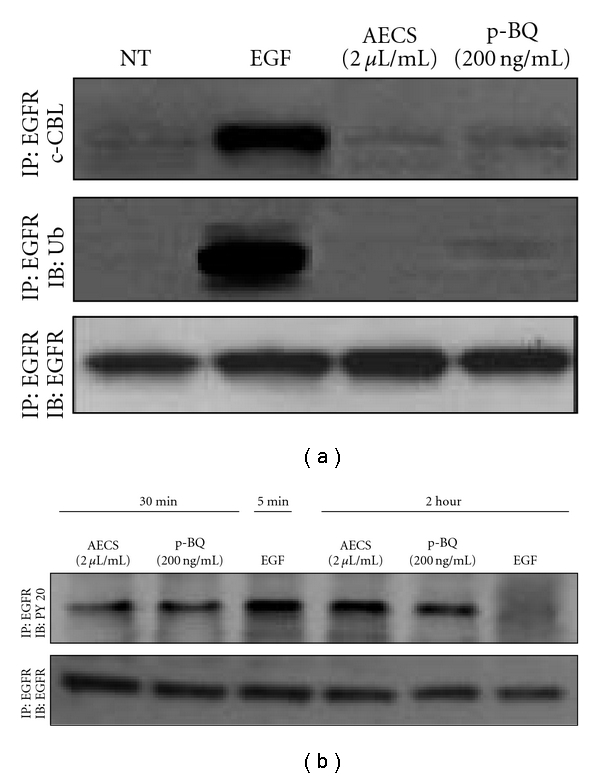
EGFR exposed to AECS/p-BQ cannot bind c-Cbl and is not ubiquitinated. Serum-starved A549 cells were either nontreated (NT) or exposed to 100 ng/mL EGF for 5 min (used as positive control), 2 *μ*L/mL AECS or 200 ng/mL p-BQ for 1 hr. After treatment, cells were immediately lysed, and the EGFR was immunoprecipitated (IP) from the cell lysates using anti-EGFR antibody. Immunoprecipitated proteins were separated by SDS-PAGE, transferred to PVDF membrane, and immunoblotted (IB) with the indicated antibodies (a). Serum-starved A549 cells were either nontreated (NT) or exposed to 100 ng/mL EGF for 5 min or 2 hr, 2 *μ*L/mL AECS or 200 ng/mL p-BQ for 1 hr. After treatment, cells were washed with PBS and further incubated in fresh serum-free medium for 2 hr at 37°C before lysis. The EGFR was immunoprecipitated (IP) from the cell lysates using anti-EGFR antibody. Immunoprecipitated proteins were separated by SDS-PAGE, transferred to PVDF membrane, and immunoblotted (IB) with antiphosphotyrosine (PY20) antibody (b).

c-Cbl (120 kDa) is an E3 ubiquitin ligase that plays a crucial role in downregulating the EGFR. On EGFR activation, c-Cbl associates with phosphorylated Tyr-1045 and ubiquitinates the receptor, marking it for clathrin-mediated endocytosis and recognition by the lysosomal machinery, which results in receptor degradation and signal termination [37–40]. EGFR exposed to 100 ng/mL EGF, its cognate ligand, is associated with c-Cbl and ubiquitinated (Figure 9(a)). However, EGFR exposed to 2 *μ*L/mL AECS for 1 hr or 200 ng/mL p-BQ for 1 hr is not phosphorylated on Tyr-1045, which renders it unable to associate with c-Cbl and precludes it from being ubiquitinated (Figure 9(a)). Therefore, under AECS/p-BQ exposure, c-Cbl loses its ability to bind to EGFR and thereby lacks ubiquitination and degradation, which leads to prolonged signaling even after removal of the external stimuli AECS/p-BQ. 

In order to study the fate of EGFR after incubation with AECS/p-BQ followed by removal of these ligands, we continued incubation of the cells for 2 hr more in serum-free medium. The results (Figure 9(b)) indicate that EGFR phosphorylation persists up to 2 hr after pretreatment with 2 *μ*L/mL AECS or 200 ng/mL p-BQ followed by removal of the ligands. This demonstrates that signals from activated EGFR prolong apparently due to the inability of EGFR degradation after AECS/p-BQ exposure. On the other hand, when the cells were pretreated with 100 ng/mL EGF for 5 min followed by removal of EGF, EGFR phosphorylation does not prolong more than 15 min [29]. Here we show that phosphorylation of EGFR by EGF is practically nil after 2 hr (Figure 9(b)).

### 3.8. AECS/p-BQ Exposure Activates Hras + Kras Which Leads to Downstream Survival and Proliferative Signaling ERK 1/2 and Akt

**Figure 10:**
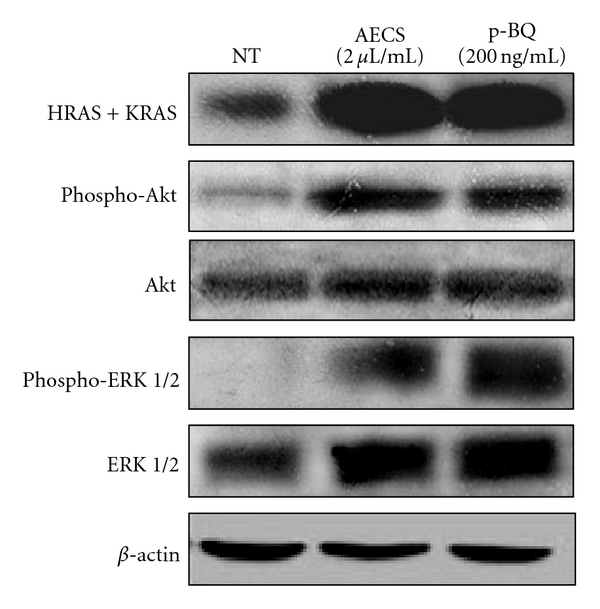
Exposure of A549 cells to AECS/p-BQ results in overexpression of Hras + Kras and activation of downstream proliferation and survival signaling Akt and ERK 1/2. Serum-starved A549 cells were either nontreated (NT) or exposed to 2 *μ*L/mL AECS or 200 ng/mL p-BQ for 1 hr followed by incubation in serum containing media for 6 hr. After 6 hr, cells were lysed, and cell lysates were separated by SDS-PAGE, transferred to PVDF membrane, and immunoblotted with antiphosphotyrosine (PY20), anti-HRAS + KRAS, anti-phospho-Akt, anti-Akt, anti-phospho-ERK 1/2, and anti-ERK 1/2 antibodies. *β*-actin was used as the loading control.

The Ras proteins are GDP/GTP-binding proteins that act as intracellular signal transducers. The inactive forms are GDP bound. They are activated by receptor tyrosine kinases including EGFR. The most well studied members of the *ras* gene family are* Hras* and *Kras*. These genes encode immunologically related proteins with a molecular mass of 21 kDa and are homologs of rodent sarcoma virus genes that have transforming abilities. While these wildtype cellular proteins in humans play a vital role in normal tissue signaling, including proliferation, differentiation, and senescence, mutated or overexpressed genes are potent oncogenes that play a major role in many human cancers including lung cancer. Here, we show that exposure to 2 *μ*L/mL AECS or 200 ng/mL p-BQ for 1 hr results in the overexpression of Hras + Kras proteins (Figure 10, row 1).

In addition to Hras and Kras, two well-established mediators of proliferation and cell survival, extracellular signal-regulated kinase (ERK1/2), also called the mitogen-activated protein kinase (MAPK), and Akt (also known as protein kinase B), are known to be involved in cell transformation when persistently activated [41–43]. It is known that activation (phosphorylation) of the ERK1/2 pathway is involved in malignant transformation both *in vitro* and *in vivo.* It is also reported that activation of ERK 1/2 is associated with nonsmall cell lung cancer (NSCLC), 80% of which is caused by cigarette smoking [44]. ERK 1/2 is activated by dual phosphorylation on both Thr202 and Tyr204 residues. Akt is a serine/threonine protein kinase that plays a key role in multiple cellular processes, including promotion of cell survival in several cell lines. Activation of both ERK 1/2 and Akt in a variety of cells is mediated mainly by growth factor receptors that require EGFR phosphorylation [45]. Furthermore, lack of EGFR turnover has been shown to mediate tumor promotion in nonneoplastic rat liver epithelial cells [46]. Figure 10 (rows 2 and 4) shows that exposure to 2 *μ*L/mL AECS or 200 ng/mL p-BQ for 1 hr results in the phosphorylation of downstream ERK 1/2 and Akt signals that render them active. However, the nontreated cells do not show any overexpression of activated Hras + Kras, ERK 1/2, or Akt (Figure 10, rows 3 and 5). 

### 3.9. AECS/p-BQ Exposure Results in Activation of c-Myc and Overexpression of c-Fos

The c-Myc protein (49kDa) is a transcription factor, which is encoded by the *c-Myc* gene on human chromosome 8q24. The c-Myc oncoprotein is among the most potent transforming agents in human cells. Elevated levels of the c-Myc oncoprotein contribute to the initiation and progression of most human tumors [47–49]. Increased expression of c-Myc induces proliferation and inhibits differentiation. c-Myc is commonly activated in a variety of tumor cells and can either activate or repress the expression of specific target genes associated with various biological functions. Via this transcriptional regulatory activity, c-Myc contributes to diverse aspects of cancer biology, including cell cycle progression, angiogenesis, metastasis, cell adhesion, cell growth, and genomic instability. Studies revealed a functional association between phosphorylation of c-Myc at Thr58/Ser62 by ERK 1/2 in cell proliferation and cell cycle regulation [50]. 

**Figure 11:**
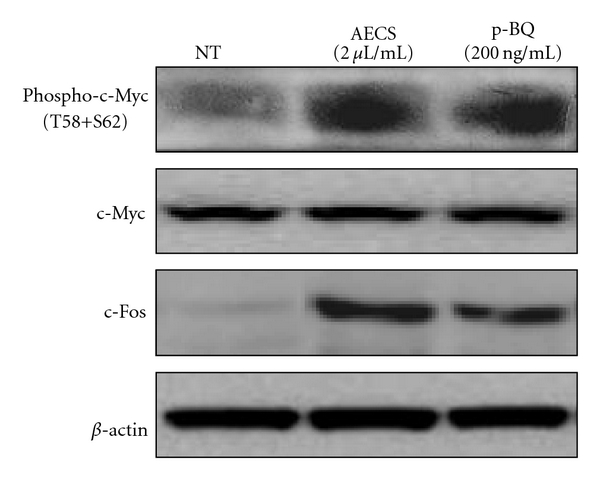
Exposure of A549 cells to AECS/p-BQ results in phosphorylation of c-Myc at Thr 58/Ser 62 and overexpression of c-Fos. Serum-starved A549 cells were either nontreated (NT) or exposed to 2 *μ*L/mL AECS for 1 hr or 200 ng/mL p-BQ for 1 hr followed by incubation in serum containing media for 6 hr. After 6 hr, cells were lysed, and cell lysates were separated by SDS-PAGE, transferred to PVDF membrane, and immunoblotted with anti-phospho-c-Myc (phospho Thr58 + Ser62), anti-c-Myc, and anti-c-Fos antibodies. *β*-actin was used as loading control.

*c-Fos* belongs to the Fos family of nuclear oncogenes. The expression of c-Fos protein (62 kDa) is rapidly and transiently induced by a variety of extracellular stimuli, including growth factors. In addition to increased expression, phosphorylation of Fos proteins by ERK kinases in response to external stimuli may further increase transcriptional activity [51–54]. Deregulated expression of c-Fos can result in neoplastic cellular transformation [51].  Figure 11 shows that exposure of A549 cells to 2 *μ*L/mL AECS or 200 ng/mL p-BQ for 1 hr results in phosphorylation of c-Myc protein at Thr58/Ser62 and overexpression of c-Fos. However, the nontreated cells neither show any c-Myc phosphorylation nor c-Fos expression.

## 4. Conclusion

**Figure 12:**
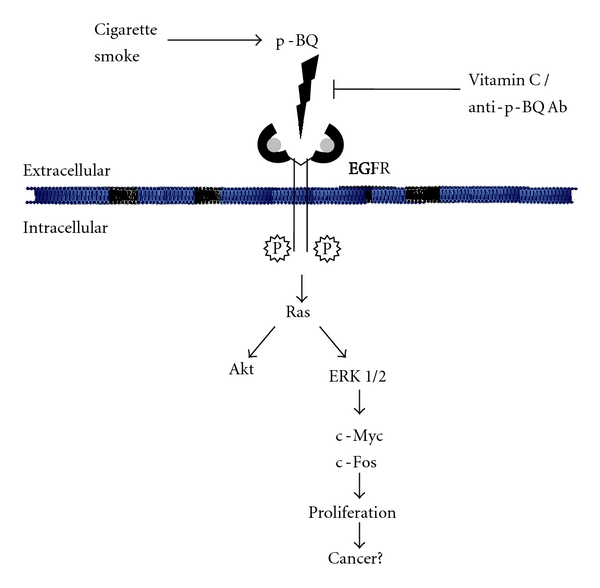
Model showing the molecular mechanisms of cigarette smoke-induced proliferation of human lung epithelial cells (A549) and prevention by vitamin C as well as antibody to p-Benzoquinone.

Despite major advances in the treatment and management of lung cancer, most patients with lung cancer eventually die of this disease. Because conventional therapies have failed to make a major impact on survival, newer approaches are necessary in the battle against lung cancer [55]. It is known that cigarette smoking is the strongest risk factor for developing lung cancer. Eventually the best method to prevent lung cancer is cessation of smoking, which has proven difficult to achieve and unlikely to be accomplished. This has motivated an intense interest in the chemoprevention of this disease [56]. Cigarette smoke (CS) is a complex mixture of about 4000 compounds [11, 12], and identifying the risk factor in CS is essential for achieving this goal. We have identified p-benzoquinone (p-BQ) as a risk factor that is tentatively produced from p-benzosemiquinone [13–15] of aqueous extract of CS (AECS). Lung cancer is believed to arise after a series of progressive pathologic changes (preneoplastic lesions) that are initiated by proliferation [4]. We have shown that low concentration of AECS or equivalent amount of p-BQ derived from AECS causes excessive proliferation of human lung epithelial cells (A549) that is mediated via aberrant phosphorylation of EGFR resulting in persistent activation of EGFR. The prolonged activation of EGFR is accompanied by activation of Ras, the downstream survival, and proliferative signaling molecules Akt and ERK1/2, as well as the transcription factors c-Myc and c-Fos. Given that more than 80% of CS-induced nonsmall cell lung carcinomas (NSCLCs) express EGFR [2], inhibition of EGFR has become an important therapeutic target for the treatment of these tumors [28, 57, 58]. We have demonstrated that both anti-p-BQ antibody and vitamin C prevent AECS/p-BQ-induced activation of EGFR and proliferation of lung cells (Figure 12). Vitamin C prevents AECS/p-BQ-induced proliferation apparently by reducing and thereby inactivating p-BQ. We consider that prevention of CS-induced proliferation of lung cells by vitamin C and/or anti-p-BQ antibody may provide a novel intervention for preventing initiation of CS-induced lung cancer.
